# Systemwide Clinical Ultrasound Program Development: An Expert Consensus Model

**DOI:** 10.5811/westjem.2018.4.37152

**Published:** 2018-06-05

**Authors:** Robert Strony, Jennifer R. Marin, John Bailitz, Anthony J. Dean, Mike Blaivas, Vivek Tayal, Chris Raio, Rachel Liu, Aimee Woods, Michael Zwank, Matthew Fields, Alyssa Abo, Stan Wu, Tarina Kang, Teresa Liu, Megan Leo, Courtney Smalley, Jerry Chiricolo, Mikaela Chilstrom, Resa E. Lewiss

**Affiliations:** * Geisinger Health Systems, Department of Emergency Medicine, Danville, Pennsylvania; † University of Pittsburgh School of Medicine, Departments of Pediatrics and Emergency Medicine, Pittsburgh, Pennsylvania; ‡ Northwestern University Feinberg School of Medicine, Department of Emergency Medicine, Chicago, Illinois; § University of Pennsylvania, Department of Emergency Medicine, Philadelphia, Pennsylvania; ¶ University of South Carolina School of Medicine, Department of Emergency Medicine Piedmont Hospital, Newnan, Georgia; || Carolinas Medical Center, Department of Emergency Medicine, Charlotte, North Carolina; # Good Samaritan Hospital Medical Center, Department of Emergency Medicine, Los Angeles, California; ** Yale University School of Medicine, Department of Emergency Medicine, New Haven, Connecticut; †† INOVA Fairfax Hospital, George Washington University, Department of Emergency Medicine, Falls Church, Virginia; ‡‡ Regions Hospital, Department of Emergency Medicine, St. Paul, Minnesota; §§ Kaiser Permanente, Department of Emergency Medicine, San Diego, California; ¶¶ Children’s National Medical Center, George Washington University School of Medicine and Health Sciences, Departments of Pediatrics and Emergency Medicine, Washington District of Columbia; |||| Baylor College of Medicine, Department of Emergency Medicine, Houston, Texas; ## Keck School of Medicine, Associate Professor of Clinical Emergency Medicine, Los Angeles, California; *** Harbor-UCLA Medical Center, Department of Emergency Medicine, Torrance, California; ††† Boston Medical Center, Boston University School of Medicine, Department of Emergency Medicine, Boston, Massachusetts; ‡‡‡ Cleveland Clinic, Department of Emergency Medicine, Cleveland, Ohio; §§§ New York Methodist Hospital, Department of Emergency Medicine, Brooklyn, New York; ¶¶¶ Emory University School of Medicine, Department of Emergency Medicine, Atlanta, Georgia; |||||| Thomas Jefferson University Hospital Department of Emergency Medicine, Philadelphia, Pennsylvania

## Abstract

Clinical ultrasound (CUS) is integral to the practice of an increasing number of medical specialties. Guidelines are needed to ensure effective CUS utilization across health systems. Such guidelines should address all aspects of CUS within a hospital or health system. These include leadership, training, competency, credentialing, quality assurance and improvement, documentation, archiving, workflow, equipment, and infrastructure issues relating to communication and information technology. To meet this need, a group of CUS subject matter experts, who have been involved in institution- and/or systemwide clinical ultrasound (SWCUS) program development convened. The purpose of this paper was to create a model for SWCUS development and implementation.

## INTRODUCTION

Clinical ultrasound (CUS) is integral to the practice of an increasing number of medical specialties. CUS significantly augments the accuracy and timeliness of many aspects of patient care, including diagnosis, monitoring, and procedural guidance.^1^–^19^ Health systems have identified a need to establish a systemwide clinical ultrasound (SWCUS) program. As a result, many emergency physicians are being tasked with leading these programs and initiatives at their health systems.

Guidelines are needed to ensure effective CUS utilization across health systems and to support consistent and high-quality CUS utilization across the range of clinical settings in which it is used. Such guidelines should address all aspects of CUS within a hospital or health system. These include leadership, training, competency, credentialing, quality assurance and improvement, documentation, archiving, workflow, equipment, and infrastructure issues relating to communication and information technology. To our knowledge, no literature addresses this specific topic. The purpose of this paper was to create a model for SWCUS development and implementation.

## METHODS

This paper is an expert consensus opinion and descriptive model. No research was performed. We queried Medline/PubMed using the keywords: System-Wide Clinical Ultrasound Director, System-Wide Clinical Ultrasound Initiative, Point-of-Care Ultrasound Director and Point-of-Care Ultrasound Initiative. No direct and relevant articles were found. Because of the lack of peer-reviewed data pertaining to this concept, we created a consensus writing group comprised of emergency medicine subject matter experts. All related references were vetted and reviewed by two authors (RS, JM). Disagreements were discussed. A group of SWCUS subject matter experts from the American College of Emergency Physicians Ultrasound Section (ACEP US), Society of Clinical Ultrasound Fellowships (SCUF) and Academy of Emergency Ultrasound (AEUS) of the Society for Academic Emergency Medicine, who have been directly involved in institution and/or SWCUS program development, was convened. We used in-person meetings, teleconferences, online sharing software, and email communications to create a model for a SWCUS program. Because this was not a research study, the initiative was exempt from the institutional review board.

### Systemwide CUS Director and Committee

The mission of a SWCUS program is to collaborate with departments using CUS to improve patient care and standardize CUS across the health system. The organizational purview of a SWCUS program includes but is not limited to the following: initial training, continuing education, credentialing, documentation, archiving, reimbursement, workflow solutions, equipment purchasing, and quality assurance and improvement. Such responsibilities are likely to increase as CUS utilization spreads within specialty-practice domains and increases among individual providers. An effective SWCUS program requires a director, with experience in interdisciplinary and interdepartmental team building, leadership, and technical expertise in CUS. In most settings, it is anticipated that the director will be the head of a SWCUS committee. The SWCUS committee should include CUS leaders from all departments and divisions across the health system that either use CUS or are involved in any of the administrative aspects of the program. This may also include team members from information technology, information security systems, revenue capture, clinical engineering, and infection control. To be effective, support is also needed from the executive leaders within the health system, such as hospital chief executive, medical, and information officers, participating clinical and ancillary department chairs, as well as the executive medical staff board and system credentialing committee, or equivalent.

In most cases, the SWCUS director will be appointed by, and report to, the chief medical officer and the executive medical staff board. The ability to effectively discharge the responsibilities summarized in the Table requires at minimum 0.5 full-time equivalent. This varies based upon the health system size, CUS utilization, and other responsibilities. As it expands, a SWCUS program is likely to require other resources (apart from the capital and infrastructure costs of performing clinical ultrasonography) such as those for clerical and administrative staff, and office space.

### Competency and Training

CUS competency assessment is a necessity for all participating medical specialties 20–23 and is increasingly being introduced at the medical school level.^24^–^27^ SWCUS leaders are able to coordinate knowledge and skills training for numerous departments, thereby reducing redundant efforts and overall teaching hours by any individual faculty or department.

As CUS is adopted by new medical specialties and its applications within medical specialties are extended, new ultrasound educational programs will need development. This role will naturally fall to the SWCUS director and team. SWCUS leadership will be able to collaborate with departments and divisions newly adopting ultrasound practices to ensure that their standards and workflow reflect institutional guidelines. Institutionally-developed training resources such as curricula and lectures can be redeployed to minimize the workload of new educational initiatives. Other medical professionals including nurses, advanced practice providers, intravenous technicians, anesthesia personnel, and prehospital teams may also need CUS training. CUS leadership can help to coordinate such education synergistically with other programs.

### Credentialing

Individual departments using CUS and the credentialing committees are typically responsible for ensuring compliance with national and local standards and with specialty-specific CUS training and credentialing policies. SWCUS leadership should be of assistance in coordinating credentialing policies that are consistent across the institution. Creation of an institutional credentialing policy can assist departments lacking formal, specialty-specific CUS guidelines and provide practice-based pathways for physicians seeking CUS but lacking previous training. All clinicians seeking credentialing in CUS should demonstrate at a minimum, the following knowledge:

- Basic ultrasound physics
- Operation of basic machine controls (e.g., depth, zoom, gain, focus, image capture)
- Image optimization
- Relevant normal and abnormal sonographic anatomy and physiology
- Biosafety
- Specialty-specific scope of CUS applications and limitations

The SWCUS director should be an active member of committees within the health system that oversee CUS credentialing to ensure a clinician applying for CUS privileging meets the institutional requirements for CUS.

### Quality Assurance and Improvement

In accordance with existing specialty-specific guidelines, individual department CUS leadership should be responsible for timely, quality assurance review of CUS examinations and providing feedback to their clinicians.^2^,^29^ SWCUS leadership should be responsible for ensuring that effective quality planning, quality assurance and continuous quality improvement processes are used across all departments. This includes regular review of department- and division-level training, credentialing, competency assessment, documentation, and oversight review of adverse outcomes potentially related to CUS. The SWCUS leadership should participate with the institutional oversight committee in any adverse outcome analysis or root cause analysis related to CUS.

### Documentation, Archiving Workflow Solutions, and Reimbursement

SWCUS leadership should work with individual departments to ensure proper documentation and image archiving. Medical record documentation of CUS should comply with institutional, local, regional, and national standards.^4^ SWCUS leadership should ensure that CUS images and interpretations performed as part of patient care are readily available to other clinicians, either through the health system’s picture archiving and communications system (PACS) or a vendor neutral archive (VNA) consistent with other institutional practices and standards.

Extensive CUS reimbursement guidelines have been published.^2^,^30^–^34^ The SWCUS director should ensure that CUS reimbursement practices are consistent throughout the institution and integrated with the reimbursement practices of traditional imaging specialists. SWCUS leaders will coordinate clinical departments, hospital information technology, and billing departments to implement CUS workflow solutions that promote efficiency and quality care, and meet standards of meaningful use. At the minimum, an integrated SWCUS workflow solution should include the following:

- Ability to generate a CUS report (either at the point of care or through accessing a server)
- Wireless (preferred) transfer of CUS images to a server or cloud for quality review and archival
- Wireless transfer of CUS images to a hospital PACS with image interpretation report in the electronic medical record
- Ability to de-identify images and videos that are used for teaching and education
- Capacity for storage of educational/practice ultrasound examinations in a location that is separate and different from the CUS evaluations that are part of medical decision-making
- Ability to flag CUS examinations for future query (e.g., teaching, research, follow-up)
- Ability to generate billing reports that can be accessed by billing departments, thus facilitating accurate and consistent billing of these examinations

### Equipment Purchasing and Maintenance

CUS equipment needs vary among specialties and practice settings. In addition, there is continual technical and ergonomic improvement in ultrasound equipment. Purchasing decisions should be made by SWCUS leadership in collaboration with the clinicians using ultrasonography in their practice.

Important factors to consider include image quality, transducer options, advanced software packages, user interface, educational support, durability, warranty, expected costs, machine size, medical record and workflow solution integration.^35^–^36^ Individual departments may have unique equipment needs based on the type and volume of CUS examinations performed. It is ideal to have the SWCUS leadership coordinate real-time equipment demonstrations from key vendors. In addition, with the advent of pocket-size ultrasound machines on tablets or phone-size devices, it is incumbent on the health system to provide guidance for purchase, security, image transmission, and maintenance.

Standardization of equipment across a healthcare system has advantages and disadvantages. Advantages include clinician familiarity, simplified integration with the electronic medical record, uniform workflow solutions, and the possibility of bulk pricing for purchases, upgrades and repairs. Disadvantages include the significantly increased costs of replacing equipment on a system-wide basis if it becomes apparent that more competitive alternatives exist, and the lack of specialty-specific capabilities of some ultrasound equipment.

Several strategies exist to reduce equipment costs, while allowing application and specialty-specific needs within the system. As noted, “bulk” purchasing may afford significant cost savings because many manufacturers provide discounts based on number of systems purchased. SWCUS leadership might also facilitate purchasing by improving revenues for CUS services, by increasing the efficiency and decreasing the redundancy of CUS services throughout the institution, and by applying for non-departmental discretionary institutional funds. Leadership and knowledge of various hardware, software and ultrasound applications will be needed.

## FUTURE DIRECTIONS AND BARRIERS

With the increasing use of CUS across many medical specialties, it is important for hospitals and hospital systems to ensure standardized, accurate, safe and responsible utilization of this important diagnostic and procedural modality. This expert, consensus-based document outlines the key components of a SWCUS program needed for a robust and successful program. The authors acknowledge the potential for changes in understanding as the field progresses. Barriers to establishing a SWCUS program include lack of executive leadership support, poor interdepartmental cooperation, inadequate time allocation and insufficient financial support. As SWCUS programs arise and evolve at many health systems, their impact will need to be measured. Future research should quantify the impact of a SWCUS program on health system quality of care, patient safety and cost savings. Downstream benefits of a SWCUS program such as improvements in clinical competency, workflow integration and interdepartmental team building should also be investigated.

## Figures and Tables

**Table:** Responsibilities of the director of a systemwide clinical ultrasound CUS program.

Oversight of CUS committee, and execution and implementation of its actions.
Oversight of training, continuing education, and credentialing across disciplines
Quality review and improvement across CUS disciplines
Documentation, archiving, reimbursement, and workflow solutions
Equipment purchase and other capital and infrastructure expenditures
